# Cardiovocal Syndrome Secondary to an Aortic Aneurysm

**DOI:** 10.1155/2016/9867942

**Published:** 2016-03-20

**Authors:** Hsing-Won Wang, Mei-Chien Chen, Pin-Zhir Chao, Fei-Peng Lee

**Affiliations:** ^1^Department of Otolaryngology, Taipei Medical University, Shuang-Ho Hospital, No. 291, Jhong-Jheng Road, Jhonghe District, New Taipei City 23561, Taiwan; ^2^Graduate Institute of Clinical Medicine, College of Medicine, Taipei Medical University, Taipei City, Taiwan; ^3^Department of Preventive and Community Medicine, Taipei Medical University, Shuang-Ho Hospital, No. 291, Jhong-Jheng Road, Jhonghe District, New Taipei City 23561, Taiwan

## Abstract

We reported that a 68-year-old man presented to the ENT outpatient department complaining of hoarseness for more than 10 months. Clinical exam identified left vocal palsy in the paramedian position and atrophic vocal folds were noted. Chest radiography revealed a large bulging contour overlying aorta and left hilar shadow. Aortic aneurysm was proved by CT scanning. Contrast-enhanced chest computed tomography for further evaluation showed a broad-based aortic aneurysm at proximal descending aorta, projecting anterolaterally. Cardiovocal syndrome was proved. The syndrome is a rare clinical presentation. While a patient with unilateral vocal palsy is encountered, one might keep in mind the possibility of cardiovocal syndrome especially in an adult who had a cardiovascular disease.

## 1. Introduction

Nobert Ortner first described hoarseness, which resulted from left recurrent laryngeal nerve palsy, in three patients with severe mitral stenosis in 1897 [1]. Later in 1958, Stocker and Enterline further identified hoarseness attributable to recurrent laryngeal nerve paralysis caused by cardiovascular disease as cardiovocal syndrome [2, 3]. Cardiovocal syndrome is a rare condition characterized by hoarseness of voice associated with cardiovascular pathology. Compression of the left recurrent laryngeal nerve by the pulmonary artery or left atrium is usually responsible. There were only few individuals described within the literature identified with left-sided vocal fold paresis/paralysis associated with an aortic aneurysm [4–6]. We encountered and reported an interesting case that had husky voice for more than 10 months.

## 2. Case Report

**Figure 1:**
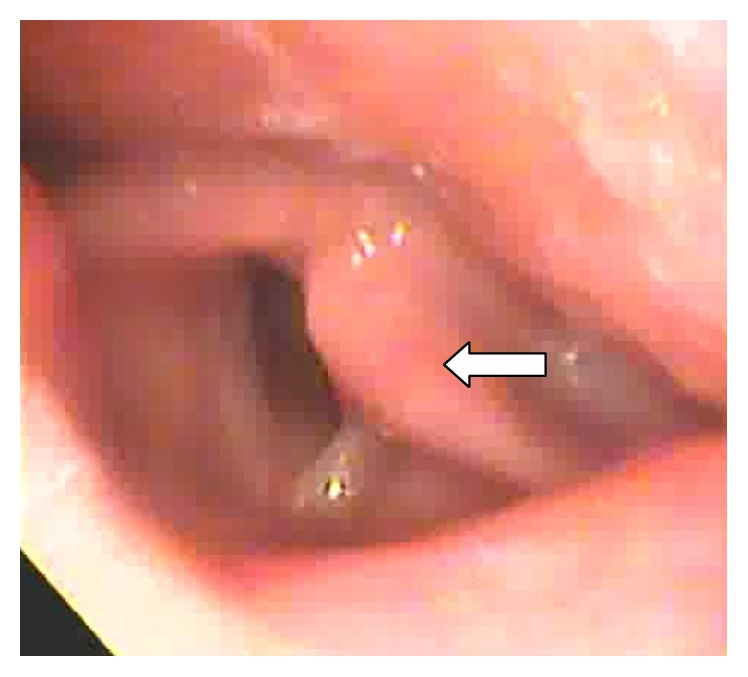
Left vocal fold fixed in abduction during respiration.

**Figure 2:**
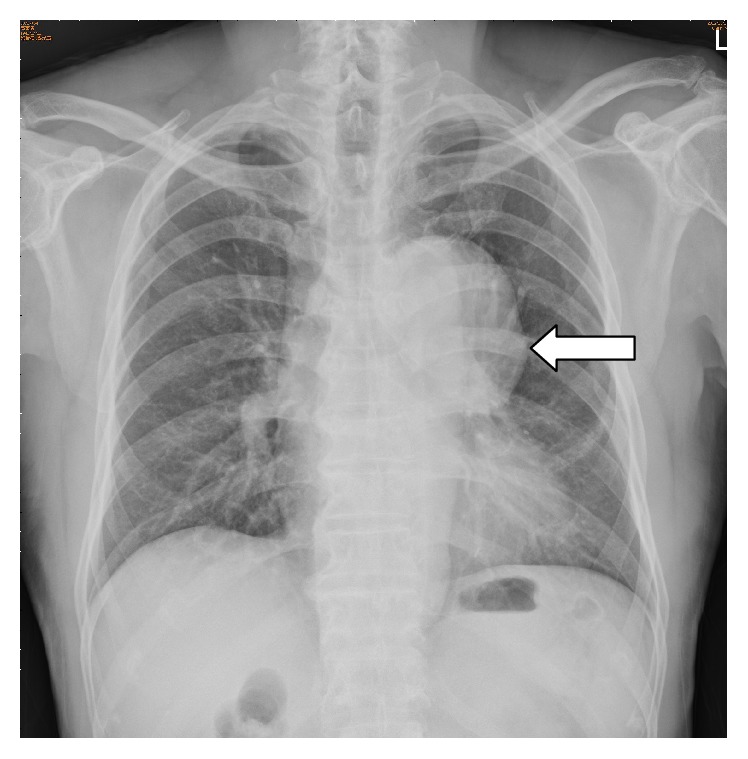
Chest radiography revealed a large bulging contour overlying aorta and left hilar shadow.

**Figure 3:**
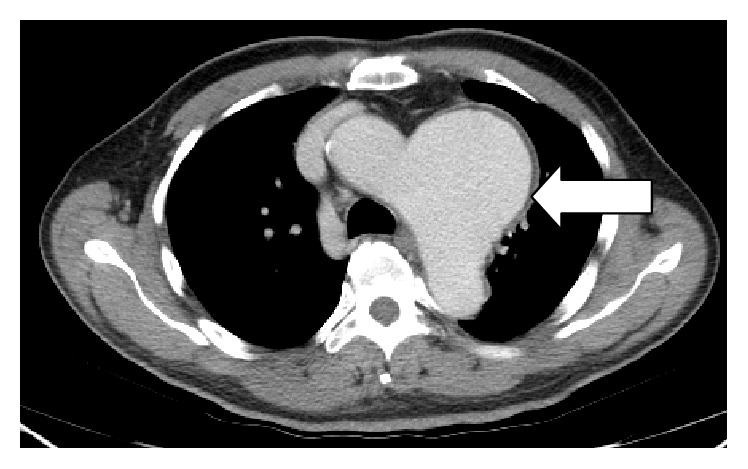
Transverse chest CT scanning, arrow indicated the aortic aneurysm.

**Figure 4:**
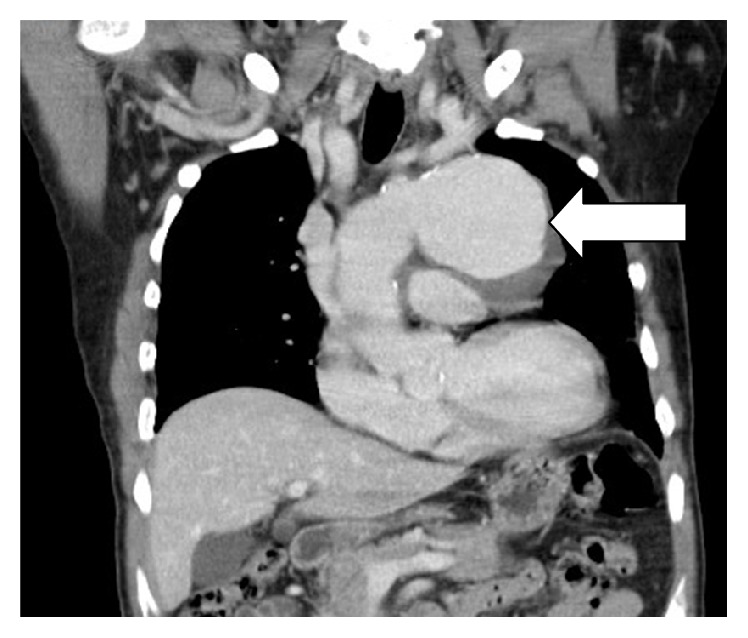
Aortic aneurysm at proximal descending aorta in coronal scanning (arrow).

A 68-year-old man presented to the ENT outpatient department complaining of hoarseness for more than 10 months. He denied symptoms of choking or dysphagia. He had a history of coronary artery bypass graft surgery 4-5 years earlier at another hospital. He had a regular follow-up in that hospital since then. Clinical examination identified left vocal palsy in the paramedian position and bilateral atrophic corditis were noted (Figure 1). A fat injection laryngoplasty was planned. However, chest radiography revealed a large bulging contour overlying aorta and left hilar shadow was noted (Figure 2). An aortic aneurysm was highly suspected. For further evaluation, the contrast-enhanced chest computed tomography showed a broad-based aortic aneurysm at proximal descending aorta, projecting anterolaterally, just distal to the left subclavian artery orifice, about 6.9 cm in largest dimension with mural thrombus (Figures 3 and 4). After explaining to the patient and his family, they decided to go to treat the aneurysm first.

## 3. Discussion

The most common cause of unilateral vocal palsy is lung cancer (42%). Iatrogenic cause (24%) comes the second. Ortner's syndrome constitutes only part of the other causes (11%) [3]. Ortner's syndrome, also known as cardiovocal syndrome, refers to hoarseness due to recurrent laryngeal nerve palsy secondary to cardiovascular disease which comprises all kinds of disease such as mitral stenosis, mitral prolapse, mitral regurgitation, pulmonary artery hypertension, aortic aneurysm, aortic dissection, pulmonary embolism, and left atrial enlargement. There are congenital causes such as atrial septal defect, ventricular septal defect, Eisenmenger's complex, and patent ductus arteriosus [7]. Initially, Ortner postulated that left recurrent laryngeal nerve was compressed by enlarged left atrium against the aorta arch. Later, Fetterolf and Norris conducted several autopsy studies and suggested that the distance between the aorta and pulmonary artery was only 4 mm, hence, most likely responsible for palsy [8, 9].

Left recurrent laryngeal nerve arises from the vagus nerve on the left of the arch of aorta, curves below it and behind the attachment of ligamentum arteriosum to the concavity of the arch and ascends in the tracheooesophageal groove where it is intimately related to the medial surface of the thyroid gland before it passes under the lower border of inferior constrictor muscle. For this case, the palsy resulted from left recurrent laryngeal nerve being compressed between the pulmonary artery and the aorta or aortic ligament as a result of enlargement of one or more of these structures due to the dilation of an aortic aneurysm. The nerve could be both stretched and compressed causing impaired function due to the enlargement of the aortic vessel at the location where this nerve resided.

There were reports of reversible nerve palsy after disease correction and there were no reports of the opposite. Due to the limitation of the number of cases, correlation of the duration of hoarseness and recovery time was not known. Generally, the degree and duration of neural damage was possibly related. This patient had an anterior cardiac surgery with orotracheal intubation and these factors might have played a role in this palsy. A coronary artery bypass graft surgery was performed 4-5 years earlier at another hospital where he had a regular follow-up. Since then no husky voice was present. He came to our ENT outpatient department complaining of hoarseness lasting about 10 months. So far it seems that the left vocal palsy with atrophic corditis was not related with both the heart surgery and the associated orotracheal intubation. Ortner's syndrome associated with an aortic aneurism might be benefitted by an open or combined open endovascular repair. For this patient, the heart disease regarding aortic aneurism was treated by another hospital and lost follow-up. Whether the operation will affect the cord fold palsy, it needs more cases analysis to clarify this question. This case was interesting for no cardiopulmonary symptoms except the unilateral vocal palsy was encountered. For the etiology of left-sided vocal fold palsy, an aortic aneurysm needs to be taken into account.

## 4. Conclusion

Cardiovocal syndrome is a rare clinical presentation. While a patient with unilateral vocal palsy is encountered, one might keep in mind the possibility of cardiovocal syndrome especially in an adult with cardiovascular disease or in an infant since the vocal palsy might be reversible after disease correction. Left-sided vocal fold palsy associated with an aortic aneurysm needs to be taken into account.
